# Supplementary material for: Changes in apparent consumption of staple food in Mexico associated with the gradual implementation of the NAFTA
Source: PLOS Glob Public Health. 2022 Nov 23;2(11):e0001144. doi: 10.1371/journal.pgph.0001144 (PMC10021749; doi:10.1371/journal.pgph.0001144)
Supplement: S1 Table — (DOCX) [file pgph.0001144.s001.docx]

**S1 Table: Weighted values of each donor unit for the synthetic control prediction.**

|  | **Synthetic controls unit weights** | | | | | | | |
| --- | --- | --- | --- | --- | --- | --- | --- | --- |
|  | **Vegetable source food** | **Fruits and vegetables** | **Pulses** | **Nuts** | **Animal-source food** | **Meat** | **Eggs** | **Milk** |
| **Country Name** |  |  |  |  |  |  |  |  |
| Argentina | -- | 0.07 | -- | 0.63 | 0.11 | 0.07 | -- | 0.34 |
| Bulgaria | -- | -- | -- | -- | 0.11 | 0.05 | 0.44 | -- |
| Cuba | 0.10 | 0.27 | 0.15 | -- | -- | 0.30 | -- | 0.28 |
| India | 0.10 | 0.23 | 0.33 | 0.01 | -- | -- | -- | 0.00 |
| Iran | -- | -- | -- | 0.05 | -- | -- | -- | 0.07 |
| Malaysia | -- | -- | -- | -- | 0.03 | -- | 0.05 | -- |
| Mongolia | -- | -- | -- | -- | 0.02 | -- | -- | -- |
| Paraguay | -- | -- | 0.52 | -- | -- | -- | -- | -- |
| South Africa | -- | 0.43 | -- | 0.06 | -- | 0.12 | -- | 0.05 |
| Turkey | 0.49 | -- | -- | 0.03 | 0.08 | 0.08 | -- | -- |
| Venezuela | 0.22 | -- | -- | -- | 0.30 | -- | -- | -- |
| China | 0.09 | -- | -- | 0.22 | 0.36 | 0.39 | 0.51 | 0.27 |

*The donor pool included originally 55 middle-income countries. This table shows only the units that contributed to the construction of the synthetic control.
