# Supplementary material for: Changes in apparent consumption of staple food in Mexico associated with the gradual implementation of the NAFTA
Source: PLOS Glob Public Health. 2022 Nov 23;2(11):e0001144. doi: 10.1371/journal.pgph.0001144 (PMC10021749; doi:10.1371/journal.pgph.0001144)
Supplement: S2 Table — a: Macroeconomic variables for Mexico and the synthetic control. b: Apparent consumption, total calories, protein, and fat (gr/capita/day) for different food groups for Mexico and the synthetic control. (DOCX) [file pgph.0001144.s002.docx]

**S2a Table: Macroeconomic variables for Mexico and the synthetic control.**

|  |  | **synthetic controls** | | | | | | | |
| --- | --- | --- | --- | --- | --- | --- | --- | --- | --- |
| **Predictor variables** | **Mexico** | **Vegetable source food** | **Fruits and vegetables** | **Pulses** | **Nuts** | **Animal source food** | **Meat** | **Eggs** | **Milk** |
| Rural population (%) | 33.40 | 47.87 | 48.99 | 59.54 | 35.35 | 46.93 | 53.22 | 59.90 | 41.34 |
| CO2 emissions (metric tons per capita) | 3.46 | 2.70 | 4.75 | 0.83 | 3.44 | 3.86 | 3.29 | 4.78 | 3.15 |
| Primary school enrollment (%) | 114.32 | 104.34 | 95.94 | 97.50 | 107.57 | 107.99 | 109.01 | 108.78 | 108.70 |
| Life expectancy at birth (years) | 67.00 | 62.85 | 62.40 | 63.84 | 67.29 | 67.27 | 67.33 | 68.62 | 68.48 |
| Gross domestic product (US$ million dollars) | $169,000 | $89,800 | $85,200 | $66,400 | $123,000 | $117,000 | $119,000 | $129,000 | $109,000 |

Source: The World Bank

**S2b Table: Apparent consumption, total calories, protein, and fat (gr/capita/day) for different food groups for Mexico and the synthetic control.**

|  |  | **synthetic controls** | | | | | | | |
| --- | --- | --- | --- | --- | --- | --- | --- | --- | --- |
| **Predictor variables** | **Mexico** | **Vegetable source food** | **Fruits and vegetables** | **Pulses** | **Nuts** | **Animal source food** | **Meat** | **Eggs** | **Milk** |
| **Total calories** | 2882.13 | 2934.00 | 2697.55 | 2420.38 | 2884.56 | 2643.18 | 2682.72 | 2790.38 | 2747.07 |
| **Vegetable-source food** |  |  |  |  |  |  |  |  |  |
| protein | 10.88 | 10.40 | -- | -- | -- | -- | -- | -- | -- |
| fat | 1.83 | 1.94 | -- | -- | -- | -- | -- | -- | -- |
| **Fruits and vegetables** |  |  |  |  |  |  |  |  |  |
| protein | 3.74 | -- | 3.92 | -- | -- | -- | -- | -- | -- |
| fat | 3.47 | -- | 2.26 | -- | -- | -- | -- | -- | -- |
| **Pulses** |  |  |  |  |  |  |  |  |  |
| protein | 8.37 | -- | -- | 8.26 | -- | -- | -- | -- | -- |
| fat | 0.79 | -- | -- | 0.82 | -- | -- | -- | -- | -- |
| **Nuts** |  |  |  |  |  |  |  |  |  |
| protein | 0.00 | -- | -- | -- | 0.00 | -- | -- | -- | -- |
| fat | 0.47 | -- | -- | -- | 0.47 | -- | -- | -- | -- |
| **Animal-source food** |  |  |  |  |  |  |  |  |  |
| protein | 24.62 | -- | -- | -- | -- | 24.90 | -- | -- | -- |
| fat | 26.07 | -- | -- | -- | -- | 25.82 | -- | -- | -- |
| **Meat** |  |  |  |  |  |  |  |  |  |
| protein | 11.62 | -- | -- | -- | -- | -- | 11.82 | -- | -- |
| fat | 16.09 | -- | -- | -- | -- | -- | 16.00 | -- | -- |
| **Eggs** |  |  |  |  |  |  |  |  |  |
| protein | 2.31 | -- | -- | -- | -- | -- | -- | 2.27 | -- |
| fat | 1.99 | -- | -- | -- | -- | -- | -- | 2.02 | -- |
| **Milk** |  |  |  |  |  |  |  |  |  |
| protein | 8.75 | -- | -- | -- | -- | -- | -- | -- | 8.74 |
| fat | 7.67 | -- | -- | -- | -- | -- | -- | -- | 7.75 |

Source: Food Balance Sheets of the Food and Agriculture Organization of the United Nations.
