# Supplementary material for: Changes in apparent consumption of staple food in Mexico associated with the gradual implementation of the NAFTA
Source: PLOS Glob Public Health. 2022 Nov 23;2(11):e0001144. doi: 10.1371/journal.pgph.0001144 (PMC10021749; doi:10.1371/journal.pgph.0001144)
Supplement: Attachment — Submitted filename: PGPH_Responses reviewers_13Sep22.docx [file pgph.0001144.s003.docx]

**Responses to reviewers**

Minor reviews: Please re-read your manuscript and correct any inconsistencies e.g., use of quotation marks. Also see some spellings e.g. line 136 and 234 the word "therefore" is incomplete.

**Response: Thank you, we corrected the inconsistencies.**

Reviewer #1: Thank you for the opportunity to review this article. The authors assessed the "relationship between trade liberalization and changes in the trend of staple food consumption using a quasi-experimental design". The articles is presented well and gives insight into an important subject, which adds value to the field. However, I have several concerns with the methodological approach which the authors employed.

**Response: Thanks for your review and comments.**

The authors modeled splines in three periods, before 1994, 1994-2007, and from 2008. Although there is possibly good rationale for using 1994 and 2008 as cut-offs, I question if this does not create artificial inflection points and thereby enforcing a linear relationship where one might not exist. The raw data for both the intervention and control show that there are non-linear patterns within the three periods while the predicted line is linear. An example of this is the apparent consumption of animal-source foods. These show an increase in both Mexico and the synthetic controls before 1994. However, the rise was steeper for Mexico than for the control, and both experienced a decline before 1994. The regression slopes to do account for this steep increase in Mexico, but instead show a steeper increase between 1994 and 2008 and do not capture the observable steep increase in the control in the same period. I question whether the model fits the data well and this can be said for other plots as well. This for me is a major drawback of the manuscript and I suspect that a different model may lead to different conclusions.

**Response: Thank you for your comment. We agree with the reviewer that there are non-linear patterns, this is the reason why we explored two inflexion points based on the gradual implementation of NAFTA which has been modeled in a similar study to evaluate the impact of NAFTA on caloric sweeteners (Unar, et al. 2019).**

**We agree that for animal-source food, we see an increasing trend before 1994 but there is no statistically significant difference in the average increase between the control group and Mexico, as shown in Table 1. Although in the graph some points for Mexico look slightly higher than the control, others are lower. The ITSA model allows to test if there is a difference in the average trend. The same can be observed between 1994 and 2008. For some food groups, the pre-intervention trend and intercept are not comparable (as shown in Table 2). If the model created artificial inflexion points, we would see this in all food groups.**

In addition, I made other comments that could assist in improving the manuscript:

1. Line 183-185: What is the implication of excluding the non-parallel groups? I’m struggling to follow what is presented in table 2. The text also does not give details of this comparison? Are you presenting regression coefficients of apparent consumption for each of the categories, comparing Mexico to the synthetic controls?

**Response:** **Thank you for your comment. In experimental designs where treatment and control groups are randomly assigned, comparability between them at baseline in basic characteristics is guaranteed. In the absence of such a design, we rely on statistical tests to check for comparability. When the baseline magnitude and trends of the outcomes of interest between a control and treatment group are not statistically different in the pre-intervention period, we expect that in the absence of a shock, such as NAFTA, the parallel trends would continue. Therefore, the difference in the outcomes between control and treatment groups after implementation can be attributed to the intervention. If the trends are not comparable, for instance, if the pre-intervention trend is steeper for the treatment group, the post-intervention difference would be upward biased. The ITSA assesses the comparability of the study groups with two parameters: the intercept and the average change for the pre-intervention period. Table 2 presents both coefficients for all food groups.**

**We added the following to address the reviewer´s comments:**

**Methods:**

**“In experimental designs where treatment and control groups are randomly assigned, comparability between them at baseline in basic characteristics is guaranteed. In the absence of such a design, we rely on statistical tests to check for comparability. When the baseline magnitude and trends of the outcomes of interest (kcal/capita/day for each food group) between a control and treatment group are not statistically different in the pre-intervention period, we expect that in the absence of a shock, such as NAFTA, the parallel trends would continue. Therefore, the difference in the outcomes between control and treatment groups after implementation can be attributed to the intervention. If the trends are not comparable, for instance, if the pre-intervention trend is steeper for the treatment group, the post-intervention difference would be upward biased.**

**The ITSA assesses the comparability of the study groups with two parameters: the intercept and the average change for the pre-intervention period.”**

**Results**

**“Table 2 shows the coefficients for the intercept and pre-intervention trend to see if Mexico and the control groups are comparable. The ITSA model test for differences in the intercept (difference in the apparent consumption of the staple food group at baseline -1970-) between Mexico and the synthetic control; and, the pre-intervention trend that is the average change per year in the apparent consumption of a staple food before 1994 in Mexico compared to the synthetic control.”**

Table 1 and S2: This may be my ignorance, but how come the school enrollment is above 100%?

**Response: Thanks for your comment. We understand that it can be confusing, we kept the name of the variable as it appears in the World Bank database. School enrollment is a rate for the total number of students enrolled in primary education, regardless of their age, expressed as a percentage of the total population of official primary school age. This indicator may be higher than 100% due to the inclusion of students older and younger than the official age, either by repeating grades or by entering early or late at that level of education.**

**We added the following where we describe the variables:**

**“School enrollment is a rate for the total number of students enrolled in primary education, calculated as a percentage of the total population of official primary school age. This indicator may be higher than 100% due to the inclusion of students older or younger than the official age”.**

Table 2: The table can do with a more self-explanatory legend and an explanation of what the values in the table are.

**Response: Thanks for your advice. We changed the table heading as follows:**

**“Table 2: Comparability between Mexico and the synthetic control by food group: differences in the intercept and pre-intervention trend (1970-1993)”**

**We also changed the term “coefficient” for “difference (Kcal/capita/day)” and added a table footnote:**

**“The intercept is the difference in the apparent consumption of the staple food group at baseline (1970) between Mexico and the synthetic control. The pre-intervention trend is the average change per year in the apparent consumption of a stable food before 1994 in Mexico compared to the synthetic control. Only food groups with non-significant differences (p>0.05) between Mexico and the control group in the two parameters were considered comparable.”**

Table S2: The table is presenting two different sets of data. A comparison of key indicators between Mexico and the synthetic controls derived for the food categories. Secondly, it shows a comparison of apparent consumption in the different food categories between Mexico and the synthetic controls. Perhaps the manuscript might benefit from separating the table into two. It took me a while to realise what was happening

**Response: Thank you. In the new version of the manuscript we split the table in two sets of variables: 1) macroeconomic variables and 2) apparent consumption for the different food groups.**
