# Supplementary material for: Changes in apparent consumption of staple food in Mexico associated with the gradual implementation of the NAFTA
Source: PLOS Glob Public Health. 2022 Nov 23;2(11):e0001144. doi: 10.1371/journal.pgph.0001144 (PMC10021749; doi:10.1371/journal.pgph.0001144)
Supplement: Attachment — Submitted filename: Responses reviewers PGPH_27Oct22.docx [file pgph.0001144.s004.docx]

Review Comments to the Author:

Reviewer #3:

**Comment-** The study attempts to answer an important research topic: Evaluate the association between the gradual implementation of NAFTA and changes in apparent consumption of staple foods in Mexico, which is especially pertinent given the health implications of these consumptions patterns, and in congruence with the WHO’s emphasis on the importance of the balance between “trade with health” as quoted by authors.
There are limitations to using the synthetic control model and to the conclusions that can be made based on it being a 'control'. These need to be objectively and adequately discussed.

**Response:** Thank you for your comment. We added the following in the discussion:

**“The results of this study should be interpreted with caution. The ideal strategy to evaluate a program is an experimental design where treatment and control groups are randomly assigned which are not plausible for policies implemented at the national level such as NAFTA. We acknowledge the limitations of using a pool of countries as control groups for comparison. The construction of a reliable synthetic control depends on the quality of the data and finding a good set of donor units can be challenging. In this study, we used data from the World Bank and FAO to create the synthetic control. Although the donor pool was made up of 55 countries, the construction of each synthetic control only considered between 3 and 7 countries that were not statistically different from Mexico in the variables used to construct this pool. In addition, using ITSA models allowed to test basic characteristics of an adequate control group: no statistical difference in the magnitude and trend pre-NAFTA between the synthetic control and Mexico. We acknowledge that even if we excluded countries with no formal trade agreements with the United States or Canada, donor units may not be completely free of any transnational trade.”**

**Comment-** An important paper and calls for further research, such as that comparing consumption of dietary patterns with other countries that have trade deals with the US and Canada, or countries that are part of other trade deals (not US and Canada), and compare and contrast those. Interesting work.

**Response: Thank you for your comment. We added the following in the conclusion:**

**“Although for this study we excluded countries with trade agreements with the US or Canada to create a pool of units as controls not exposed to the program, future studies should compare countries with trade agreements similar to Mexico to analyze if changes in consumption or dietary patterns were similar.”**
